# Achieving climate justice, safeguarding planetary health: Diagnosis and demands from next generation leaders for COP27 and beyond

**DOI:** 10.1371/journal.pgph.0001304

**Published:** 2022-11-14

**Authors:** Renzo R. Guinto, Thilagawathi Abi Deivanayagam, Paccha Turner Chuji, Azmal Hossan, Anpotowin Jensen, Laura Jung, Eric Njuguna, Rhiannon Osborne, Melvine Anyango Otieno, Ayisha Siddiqa, Amiteshwar Singh, Bernard Kato Ewekia Taomia

**Affiliations:** 1 Planetary and Global Health Program, St. Luke’s Medical Center College of Medicine-William H. Quasha Memorial, Quezon City, Philippines; 2 Sunway Centre for Planetary Health, Sunway University, Selangor, Malaysia; 3 Institute for Global Health, University College London, London, United Kingdom; 4 Lancaster Medical School, Faculty of Health and Medicine, Lancaster University, Lancaster, United Kingdom; 5 Faculty of Human, Social and Political Sciences, University of Cambridge, Cambridge, United Kingdom; 6 Department of Sociology, Colorado State University, Fort Collins, Colorado, United States of America; 7 Interdisciplinary Training, Education and Research in Food-Energy-Water Systems, Colorado State University, Fort Collins, Colorado, United States of America; 8 Department of Civil and Environmental Engineering, Stanford University, California, United States of America; 9 German Alliance on Climate Change and Health, Germany; 10 Leipzig University Hospital, Division of Infectious Diseases and Tropical Medicine, Leipzig, Germany; 11 Fridays for Future MAPA; 12 School of Clinical Medicine, University of Cambridge, Cambridge, United Kingdom; 13 The People’s Health Movement, Global and United Kingdom; 14 Planetary Health Eastern Africa Hub, Eldoret, Kenya; 15 Department of Environmental Biology and Health, University of Eldoret, Eldoret, Kenya; 16 Polluters Out and Fossil Free University; 17 Centre for Human Rights and Global Justice, New York University, New York, NY, United States of America; 18 Norwich Medical School, University of East Anglia, Norwich, United Kingdom; 19 Health for a Green New Deal, London, United Kingdom; 20 Saving Tuvalu, Funafuti, Tuvalu; PLOS: Public Library of Science, UNITED STATES

We are next generation leaders coming from the climate justice, medicine and public health, and planetary health movements, representing Most Affected People and Areas (MAPA), which are groups and territories disproportionately affected by the climate crisis – women, Indigenous Peoples, racialised people, LGBTQ+ people, young, and those facing social deprivation, and the Global South. Each time the Conference of Parties (COP) of the United Nations Framework Convention on Climate Change (UNFCCC) is held, our presence is sought, our faces shown on television, broadsheets and social media, our voices, through speeches and interventions (temporarily) projected, yet ultimately ignored, and our spaces rather assigned, mostly in side events and in the streets. Once the conference is over, our dreams and demands are almost totally and automatically forgotten, as high-profile delegates return to their countries in private jets and business class seats – and to business as usual.

For decades, the world’s governments have been negotiating about our future – without us. Haunted by past experience, there is a high risk that the upcoming COP27, which is to be hosted by Egypt in November 2022, may not be any different from the previous COPs, where excitement and ambition are instantly trumped by the power and influence of short-term interests and corporate greed. For instance, many were anticipating last year’s COP26 held in Glasgow as a possible watershed moment for climate action, as the COVID-19 pandemic provided a “sneak preview” of deleterious human consequences of shocks and stresses that crises such as the climate emergency could bring. But in the final declaration of COP26, the initial commitment to “phase out” coal-based power in major economies by 2030 and globally by 2040, became a “phase down”, and there was no commitment to phase out fossil fuels as a whole [1]. In fact, many of the countries in the Global North are not on track to meet their pledges, and are continuing to expand fossil fuel production [2]. Additionally, the commitment made in 2009 by Global North countries to pay $100 billion per year for supporting the Global South for adaptation and mitigation has failed each year [3].

## Centering health and justice

This is not how climate justice is served and not how our collective planetary health can be safeguarded for generations to come. The climate crisis – and it must be called such – is not an issue for tomorrow or ten years from now – it is already happening now. Dramatic changes in ecosystems are already causing serious human health impacts – such as undernutrition, infectious disease, and even mental distress – especially in minoritised and vulnerable communities [4]. After all, climate change is a threat multiplier, which exacerbates the chronic problems of human society like poverty, hunger, disease, pollution, gender inequality, conflict, among others. To make our vision of a healthy future a reality, decision-makers must go beyond celebrating a health pavilion at COP (like what happened last year) and instead commit to fighting for a world in which no one’s health and life is sacrificed for profit.

In addition to emphasizing health and wellbeing for all in the climate negotiations, there must also be an explicit recognition that what is happening is a mere perpetuation of the long history of colonialism globally. The latest report of the United Nations Intergovernmental Panel on Climate Change (UN IPCC) highlighted, for the first time, the central role of historical colonialism in propelling contemporary anthropogenic climate change [5]. Our current colonial capitalist world order values only extraction from people and the Earth. Meanwhile, victims of colonialism, such as the world’s Indigenous Peoples, continue to be exploited and plundered and are now battling the climate crisis on the ground. The colonial legacy continues even inside the negotiation rooms, where the interests of the powerful prevail and Indigenous and majority world voices are silenced.

## Loss and damage, and true solutions

Acknowledging the role of colonialism in driving the climate emergency and the persistent coloniality of the current climate regime is central to the realization of “loss and damage” financing, which the rich countries of the Global North continue to delay and even reject [6]. There are human consequences, including health impacts, of the climate crisis that communities cannot anymore adapt to – similar to the consequences of past colonial subjugation that cannot anymore be reversed. Northern countries, many of them perpetrators of both historic human colonization and present-day atmospheric colonization, must fulfil their responsibility and urgently provide loss and damage financing to climate-impacted countries in the Global South and Indigenous Nations. Loss and damage financing must be a separate and permanent part of the COP agenda moving forward. The next generation must keep an eye on manoeuvres that attempt to insert loss and damage financing “by stealth” into existing adaptation or mitigation financing commitments. It must also be acknowledged that holistic reparations - beyond the limitations of financing facilitated by UNFCCC - are owed to communities across the world for the traumas of colonialism, slavery and imperialism [7]. Young people must advocate for care and cooperation between countries to actualise reparative justice.

Whilst resisting the false promises of climate finance, false solutions peddled by governments and corporations must also be strongly rejected. Offsetting becomes an excuse for continuing to emit greenhouse gases and to omit the possibilities of practices and systems that maintain the delicate web of planetary regulation [8]. Goals such as “net zero by 2050” have become a distraction from the legally-binding commitments and targets set in the Paris Agreement whose deadline is 2030 [9]. Instead of serious discussions about economic redesign that can be accomplished now if there is enough will, ideas of technologies that still do not exist – such as carbon capture and storage or geo-engineering – are the ones being heavily promoted [10]. Rather than a radical restructuring of our economic and political systems into ones that solve the intertwined crises of ecological collapse, social inequality, and health injustice, “greenwashing” continues to prevail.

## For COP hosts and developing countries

Each COP, there are concerns with regard to greenwashing, lack of transparency, and suppression of critical voices. While we expect Egypt, like other past hosts, to showcase green technologies in the conference pavilion, the Climate Action Tracker continues to rate the country’s overall climate action as “Highly Insufficient” [11]. Reports of activists being imprisoned and important research banned are even more alarming, threatening discourse and dissent that are vital to democratic negotiations [12]. Coca-Cola’s sponsorship of COP27 is another sign of greenwashing, while little is known about other possible funders of this conference, including Big Oil [13]. Next year’s COP’s host, United Arab Emirates, which is one of the world’s biggest oil exporters, must be similarly scrutinized.

Meanwhile, as we remind rich Northern countries of their responsibility, developing countries in the Global South, where most of us authors do belong, must also commit to avoiding the same destructive path of development that the Western industrialised countries followed for centuries. For example, Bangladesh, which previously led the Climate Vulnerable Forum (CVF) in the international climate negotiations, continues to prioritize fossil fuels as the main source of energy [14]. The country still produces two-thirds of its power from natural gas, while the rest of its energy mix comes from coal, liquid fuel and hydropower. However, we recognize that in order to “walk the talk”, developing countries must receive greater funding for mitigation, adaptation, and loss and damage, and that the exploitation of people and natural resources in the Global South by Northern-based multinational corporations must end.

## From climate anxiety to action

Thanks to information technology, today’s youth are perhaps some of the most knowledgeable generations when it comes to the climate crisis. Their wisdom also comes from first-hand experience, causing emotional responses such as ‘climate anxiety’ as revealed in emergent studies [15]. However, at the moment, they are not given seats around the decision-making table to be able to stop these anticipated impacts from happening in the first place.

Nonetheless, young people are creatively converting their anxiety and anger into agency and action. For instance, through advocacy and awareness raising, youth movements from the climate frontlines such as Tuvali are making their stories of struggle and survival visible to the powerful. Several of us are organizing platforms such as the People’s Health Hearing, a global forum for testimonies of how extractive industries are devastating health and how communities are resisting systems of emissions and oppression. Those of us with health backgrounds are collaborating with health systems and professional societies in using the power of health evidence and their voice to drive meaningful policy change and stir climate action.

Fundamentally, urgent climate action is about fighting for our right to experience joyful, fulfilling lives. We, the next generation, hear the suffering of those most affected, from the oil spills in Ecuador to urban air pollution in London, and therefore we resist. While we laud the increasing youth engagement in the upcoming COP27, we also reiterate the urgent necessity to truly listen to our diagnoses and demands, to achieve climate justice and safeguard planetary health for the world’s children – now and in the future.
